# Supplementary material for: Rapid Molecular Diagnostic Sensor Based on Ball-Lensed Optical Fibers
Source: Biosensors (Basel). 2021 Apr 15;11(4):125. doi: 10.3390/bios11040125 (PMC8071528; doi:10.3390/bios11040125)
Supplement: Supplementary file 1 [file biosensors-11-00125-s001.zip › biosensors-1160992 supplementary/biosensors-1160992 supplementary.pdf]

biosensors

Supplemenatry

Rapid Molecular Diagnostic Sensor Based on Ball-Lensed Optical Fibers

Byungjun Park [1,2,†], Bonhan Koo [3,4,†], Jisub Kim [1], Kiri Lee [1], Hyeonjin Bang [1], Sung-Han Kim [4], Kyung Young Jhang [2,*], Yong Shin [5,*] and Seungrag Lee [1,*]

[1] Medical Device Development Center, Osong Medical Innovation Foundation, 123 Osongsaengmyung-ro, Heungdeok-gu, 28160 Cheongju-si, Korea; yachon@kbiohealth.kr (B.P.); jiseob@intekmedi.co.kr (J.K.); krlee@kbiohealth.kr (K.L.); crisenc@kbiohealth.kr (H.B.)
[2] School of Mechanical Engineering, Hanyang University, 222 Wangsimni-ro, Seongdong-gu, , Korea
[3] Department of Convergence Medicine, Asan Medical Institute of Convergence Science and Technology, Asan Medical Center, University of Ulsan College of Medicine, 88 Olympicro-43gil, Songpa-gu, Korea; qhsgksdl@ulsan.ac.kr
[4] Department of Infectious Diseases, Asan Medical Center, University of Ulsan College of Medicine, 88 Olympicro-43gil, Songpa-gu, Korea; shkimmd@amc.seoul.kr
[5] Department of Biotechnology, College of Life Science and Biotechnology, Yonsei University, 50 Yonsei Ro, Seodaemun Gu, 03722, Korea
* Correspondence: kyjhang@hanyang.ac.kr (K.Y.J.); shinyongno1@yonsei.ac.kr (Y.S.); naviman78@kbiohealth.kr (S.L.)
† These authors contributed equally to the work.

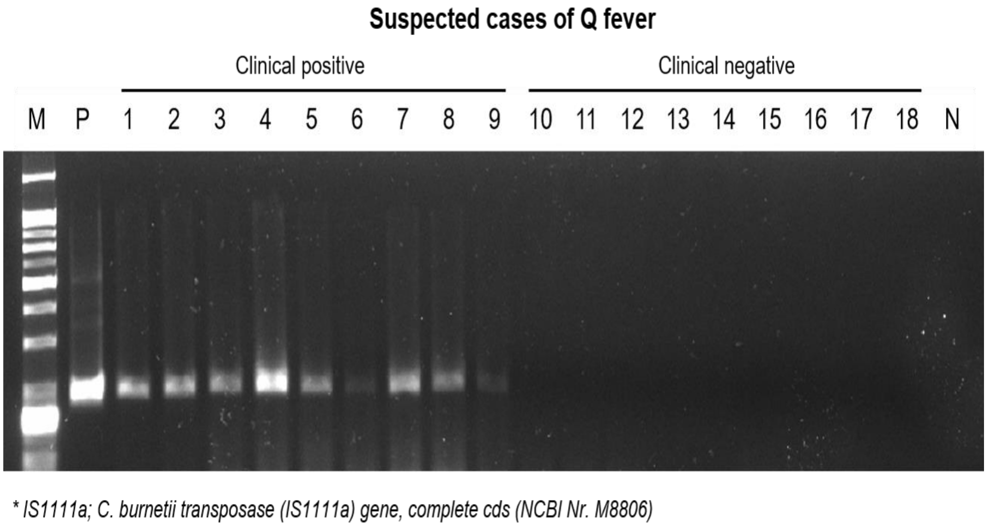

Figure S1. Gel electrophoresis results for the detection of *Coxiella burnetii* DNA in clinical specimens from patients with Q fever, using end-point PCR. (M: DNA marker; P: Positive template; 1–9: Q-fever-positive clinical specimens; 10–18: Q-fever-negative clinical specimens; N: Negative control).

Table S1. Primer sequences used for the amplification of *Coxiella burnetii* DNA.

C.burnetii	Primer		Sequence (5´ – 3´)
IS1111a	Conventional assay	Forward	GAGCGAACCATTGGTATCG
		Reverse	CTTTAACAGCGCTTGAACGT
	BLOF bio-optical sensor	Forward	NH_2-$(CH_2)_{12}$-GAGCGAACCATTGGTATCGGACGTTTATGGGGATG
		Reverse	GTATCTTTAACAGCGCTTGAACGTCTTGTTG

IS1111a; C. burnetii transposase (IS1111a) gene, complete cds (NCBI Nr. M8806)

Table S2. Simulation conditions of the ball-lensed optical fiber BLOF) with Zemax software.

**Items**		**Specifications**
**Coreless fiber (CLF)**	Coreless fiber length	285 µm
	Ball diameter	300 µm
	Cladding diameter	125 µm
**Polarization fiber (PM)**	Core diameter	8.5 µm
	Cladding diameter	125 µm
	Mode field diameter	10 µm (@1550 nm)
	Numerical aperture	0.125
**Optical Source**	Wavelength	1550 nm
